# Targeting biofilm and virulence in *Pseudomonas aeruginosa*: AidH@SPEEK as a novel quorum-sensing inhibitor

**DOI:** 10.1128/spectrum.01668-25

**Published:** 2026-03-13

**Authors:** Yiting Liu, Dongbo Li, Meng Zhou, Liru Zhao, Liujing Chen, Zhongle Cheng

**Affiliations:** 1 Department of Laboratory Medicine, the First Affiliated Hospital of Anhui Medical University, Hefei, China; Griffith University-Gold Coast Campus, Gold Coast, Australia

**Keywords:** *Pseudomonas aeruginosa*, biofilm, quorum sensing, SPEEK, AidH, infection control

## Abstract

*Pseudomonas aeruginosa* (PA), a gram-negative opportunistic pathogen, poses severe risks to immunocompromised patients. Conventional antibiotics often fail due to toxicity and resistance, primarily linked to biofilm formation controlled by quorum sensing (QS). Here, we developed an AidH-loaded sulfonated polyetheretherketone (AidH@SPEEK) composite, optimizing its loading conditions (400 μL AidH, 30 min). Enzymatic activity peaked at 40°C and pH 7.0 but remained stable at 37°C. AidH@SPEEK significantly suppressed PA biofilm formation and virulence factor secretion while downregulating QS (*LasR/LasI*, *RhlR/RhlI*) and virulence genes (*exoS*, *phzM*). Notably, it enhanced PA’s antibiotic susceptibility, offering a promising QS-targeting strategy to combat resistant infections.

**IMPORTANCE:**

The rising antibiotic resistance in *Pseudomonas aeruginosa* (PA) underscores the urgent need for alternative therapeutic approaches. This study highlights the potential of AidH@SPEEK as a non-antibiotic strategy to combat PA infections by targeting the quorum-sensing (QS) system, a key regulator of biofilm formation and virulence. By degrading QS signaling molecules, AidH@SPEEK disrupts bacterial communication, reduces pathogenicity, and enhances antibiotic sensitivity. The use of SPEEK as a delivery platform ensures sustained enzyme activity and biocompatibility, making it suitable for medical implants. These findings offer a promising direction for developing anti-infective materials that mitigate biofilm-associated resistance, ultimately improving clinical outcomes for high-risk patients.

## INTRODUCTION

*Pseudomonas aeruginosa* (PA), a gram-negative opportunistic pathogen, frequently causes severe infections in immunocompromised patients. Its high prevalence and multidrug resistance have emerged as critical global public health challenges (1, 2). According to statistics, PA infections contribute to over 500,000 patient deaths annually, with more than half of these cases closely linked to antibiotic resistance (3, 4). Traditional antibiotic therapies face limitations due to biofilm formation and quorum-sensing (QS)-mediated resistance mechanisms. Biofilms, protected by extracellular polymeric matrix barriers, reduce bacterial susceptibility to antibiotics by 10- to 1,000-fold. Concurrently, the QS system regulates virulence factor secretion and biofilm maturation through signaling pathways such as Las and Rhl, further complicating infection management (5–7).

In recent years, strategies targeting the QS system have provided novel insights for combating PA infections. PA employs N-acyl-homoserine lactones (AHLs) as QS signaling molecules to regulate gene expression, including those responsible for virulence factors (e.g., elastase, pyocyanin, and alginate) and biofilm formation (8, 9). The LasI/LasR and RhlI/RhlR systems, two coexisting regulatory circuits in PA, produce and respond to N-3-oxo-dodecanoyl-L-homoserine lactone (3-oxo-C12-HSL) (10, 11) and N-butyryl-L-homoserine lactone (C4-HSL) (12, 13). Quorum quenching (QQ), which disrupts bacterial communication by degrading AHLs, effectively suppresses virulence factor yield and biofilm development (14–16). Studies have demonstrated that AidH, a member of the α/β-hydrolase family, disrupts QS signaling by hydrolyzing the homoserine lactone ring of AHLs, significantly attenuating PA pathogenicity and enhancing antibiotic susceptibility (17). However, achieving stable immobilization and sustained release of QQ enzymes on implant surfaces remains a critical challenge in anti-infective material development.

Sulfonated polyether ether ketone (SPEEK), a high-performance biomaterial, exhibits exceptional potential for orthopedic implants due to its bone-like mechanical properties, three-dimensional porous structure, and superior drug-loading capacity (18, 19). Sulfonation enhances the hydrophilicity and cytocompatibility of PEEK, while its abundant surface sulfonic acid groups serve as an ideal platform for immobilizing bioactive molecules such as AidH (20). Despite its widespread application in medical devices (e.g., artificial joints), the insufficient antimicrobial performance of SPEEK continues to pose postoperative infection risks.

To address these limitations, this study proposes the development of a multifunctional AidH@SPEEK composite by immobilizing AidH onto SPEEK surfaces. By elucidating the enzymatic activity, biofilm-inhibitory effects, and antibiotic synergism of the composite, we aim to establish a non-antibiotic strategy for suppressing PA infections via QS disruption, thereby advancing surface-functionalized implants and offering theoretical insights for multidrug-resistant infection control.

## MATERIALS AND METHODS

### Reagents and chemicals

The following reagents were obtained from commercial suppliers: from Macklin Biochemical Co., Ltd. (Shanghai, China), azo-casein, trichloroacetic acid solution, 1,3-naphthalenediol, butyl acetate, anhydrous iron(III) chloride, crystal violet, anhydrous copper sulfate, cefepime, imipenem, meropenem, aztreonam, piperacillin, and ciprofloxacin; from Sangon Biotech Co., Ltd. (Shanghai, China), diethyl pyrocarbonate (DEPC)-treated water, SGExcel FastSYBR qPCR Master Mix, and MightyScript First-Strand cDNA Synthesis Master Mix (with genomic DNA removal); from GLPBIO Technology Inc. (Montclair, CA, USA), 4-nitrophenyl acetate and 4-nitrophenol; from Bio Froxx GmbH (Einhausen, Germany), Tris powder; from Chengdu Kelong Chemical Co., Ltd. (Chengdu, China), sodium dihydrogen phosphate, disodium hydrogen phosphate, isopropyl alcohol, and methanol; from Wuxi Look Chemical Co., Ltd. (Wuxi, China), hydrochloric acid, chloroform, and ammonium hydroxide; from Xilong Scientific Co., Ltd. (Shantou, China), absolute ethanol; from Procell Life Science & Technology Co., Ltd. (Wuhan, China), phosphate-buffered saline (PBS); from Zhejiang Apeloa Pharmaceutical Co., Ltd. (Zhejiang, China), ceftazidime powder.

### Experimental strains

PA strains were isolated from clinical sterile body fluid specimens obtained from The First Affiliated Hospital of Anhui Medical University. Following polymerase chain reaction (PCR) screening, three PA strains were selected: PA1 (resistant to third-generation cephalosporins and monobactams), PA2 (non-resistant control), and PA3 (carbapenem- and quinolone-resistant). All strains harbored four QS-related genes and two virulence genes. Additionally, *Escherichia coli* expressing AidH was kindly provided by Professor Xinjiong Fan’s research group at the School of Basic Medical Sciences, Anhui Medical University.

### Optimization of loading time and ratio for SPEEK-immobilized AidH

*Escherichia coli* expressing AidH was suspended in sodium phosphate buffer and lysed using an ultrasonic cell disruptor. The lysate was centrifuged at 12,000 rpm and 4°C for 10 min, and the supernatant containing AidH enzyme (activity: 6.84 IU/mL) was collected.

SPEEK carriers were incubated with 600 μL of AidH enzyme solution (6.84 IU/mL) under gentle agitation (150 rpm) at 4°C for varying durations (10 min to 3 h). After adsorption, the samples were transferred to 1 mL of p-nitrophenol acetate solution and reacted at 40°C for 4 min. The enzymatic activity was quantified by measuring the optical density at 405 nm OD_405_. Relative activity was calculated based on the maximum observed activity, and a time-relative enzyme activity curve was plotted.

To evaluate binding efficiency, SPEEK carriers were incubated with different volumes of AidH solution (100–1,000 μL) under identical adsorption conditions (30 min, 150 rpm). Enzymatic activity was assessed as described above, and a volume-relative enzyme activity curve was generated to determine the optimal loading ratio.

The SPEEK carrier was adsorbed with the enzyme solution (400 μL, 6.84 U/mL) at low temperature and 150 rpm for 30 min. After taking out the carrier, it was immersed in 1 mL of p-nitrophenol acetate solution and reacted in water baths at 30°C, 40°C, 50°C, 60°C, and 70°C for 4 min, respectively, and the OD_405_ value was determined. The relative enzyme activity with the maximum enzyme activity as 100% was calculated, and a line graph was drawn.

The adsorption conditions were the same as above (low temperature, 150 rpm, 30 min). The carriers were placed in substrate solutions with different pH values (5.4, 6.0, 6.4, 7.0, 7.4, 8.0, and 8.4), and OD_405_ was determined after reacting in a water bath at 40°C for 4 min. Based on the pH corresponding to the maximum enzyme activity, the relative enzyme activity was calculated, and a line graph was drawn.

The SPEEK loaded with AidH was held at 37°C and 40°C, respectively, and samples were taken at time points of 0, 1.5, 3, 6, 9, 12, 18, and 24 h. Immediately after each sampling, react in the substrate solution at 40℃ for 4 min to determine OD_405_. Taking the initial enzyme activity (0 h) as 100%, the percentage of the remaining enzyme activity was calculated, and a line graph was drawn.

### Effect of AidH@SPEEK on PA growth

PA1, PA2, and PA3 strains were streaked onto blood agar plates using the quadrant method and incubated at 37°C for 24 h. Single colonies were then inoculated into LB liquid medium and cultured at 200 rpm for 8 h.

Bacterial suspensions were diluted to an optical density of 0.1 at 600 nm (OD_600_) using fresh LB medium. Aliquots (1 mL per well) of the diluted suspensions were dispensed into a 24-well plate. For experimental groups, graded volumes (Groups 1, 2, and 3) of AidH@SPEEK were added to the bacterial suspensions, while the control group received SPEEK loaded with buffer. The plate was incubated at 37°C, and 200 μL samples were collected hourly to measure OD_600_ over 24 h. Growth curves were plotted to analyze the inhibitory effects of AidH@SPEEK on PA proliferation.

### Determination of optimal AidH@SPEEK dosage

The bacterial solution with OD_600_ = 0.1 was added to a 24-well plate at a volume of 1 mL per well. For the experimental group, AidH@SPEEK was added to the bacterial solution in equal parts 1, 2, and 3. For the control group, SPEEK loading buffer was added. The plate was incubated at 37°C for 24 h. The biofilm formation and virulence factor yield were measured to determine the final optimal addition.

### Effect of AidH@SPEEK on PA biofilm formation and virulence factor yield

#### Biofilm quantification

Biofilm formation was assessed as previously described (21). After 24 h of incubation in 24-well plates, non-adherent cells were removed by gently washing three times with PBS. The plates were air-dried at room temperature for 20 min, stained with 0.2% crystal violet for 30 min, and rinsed three times with PBS to remove residual dye. Biofilm-bound dye was solubilized in 95% ethanol for 10 min, and 200 μL of the solution was transferred to a 96-well plate for optical density measurement at OD_590_.

#### Pyocyanin measurement

Pyocyanin levels were determined following established protocols (22). Bacterial cultures were centrifuged at 13,000 rpm for 2 min, and 500 μL of supernatant was mixed with an equal volume of chloroform. After vigorous vortexing for 2 min, the organic phase was discarded, and 250 μL of 0.2 M HCl was added to the aqueous phase. The pink upper layer was analyzed at OD_520_ to quantify pyocyanin.

#### Elastase activity assay

Elastase activity was evaluated using azocasein hydrolysis (23). Briefly, 250 μL of culture supernatant was combined with an equal volume of 2% azocasein solution and incubated at 37°C for 40 min. The reaction was terminated by adding 1 mL of 10% trichloroacetic acid, followed by centrifugation at 13,000 rpm for 10 min. The supernatant was collected, and elastase activity was determined by measuring OD_415_.

#### Alginate quantification

Alginate production was measured as reported (24). A 500 μL aliquot of bacterial culture was mixed with 750 μL of 10% copper sulfate and incubated at room temperature for 1 h to precipitate alginate. After centrifugation (13,000 rpm, 10 min), the pellet was resuspended in 0.5 mL of copper-HCl reagent and 0.5 mL of naphthoresorcinol reagent. The mixture was heated at 100°C for 40 min, extracted with ethyl acetate, and centrifuged. The organic phase was analyzed at OD_565_ to determine alginate concentration.

### Effect of AidH@SPEEK on expression of quorum-sensing and virulence genes

For each PA strain (PA1, PA2, and PA3), two experimental replicates of AidH@SPEEK were co-incubated with 1 mL of bacterial suspension (OD_600_ = 0.1) in 24-well plates. Control groups consisted of two replicates of SPEEK loaded with buffer under identical conditions. All groups were incubated at 37°C for 24 h. Following incubation, bacterial cells were harvested by centrifugation (12,000 rpm, 4°C, 10 min), and total RNA was extracted using TRIzol reagent. RNA was reverse-transcribed into cDNA using the MightyScript First-Strand cDNA Synthesis Master Mix (with genomic DNA removal).

Quantitative real-time PCR (qPCR) was performed using SGExcel FastSYBR qPCR Master Mix on a StepOnePlus Real-Time PCR System. Primer sequences targeting quorum-sensing genes (*LasI*, *LasR*, *RhlI*, and *RhlR*) and virulence genes (phzM and *exoS*) were synthesized by Sangon Biotech (Shanghai, China), as previously reported (24, 25) (Table 1). Gene expression levels were normalized to the housekeeping gene 16sRNA and analyzed via the 2^−ΔΔCt^ method. Statistical significance was determined using Student’s *t*-test (*P* < 0.05).

**TABLE 1:** Primers sequence of quorum-sensing genes and virulence genes

Genes	Primer sequences (5′–3′)
*LasI*	F:CTACAGCCTGCAGAACGACA R: ATCTGGGTCTTGGCATTGAG
*LasR*	F:ACGCTCAAGTGGAAAATTGG R: GTAGATGGACGGTTCCCAGA
*RhlI*	F:TCTGGTCCAGCCTGCAATG R:TCAGCTTCTGGGTCAGCAACT
*RhlR*	F:ATGATGGCGATTTCCCCGGAAC R:CATCCGATGCTGATGTCCAACC
*exoS*	F:CGAGGTCAGCAGAGTATCGG R:GTAGAGACCAAGCGCCATCA
*phzM*	F:CCGACAACCTGGAATTGCGT R:CCGCTTTCCGTGGTCCAGTT
*16S rRNA*	F:TCTAAGGAGACTGCCGGTGA R:CAGCTGCG ATCCGGACTAC

### Combination effects of SPEEK-loaded AidH with antibiotics

#### Bacterial culture preparation

Strains PA1, PA2, and PA3 were streaked onto blood agar plates using the quadrant streaking method and incubated at a constant temperature. Single colonies were selected and inoculated into Mueller-Hinton (M-H) broth, followed by incubation at 37°C in a shaking incubator (200 rpm) for 8 h.

#### Antibiotic susceptibility testing

Bacterial suspensions from the shaking incubator were adjusted to an optical density (OD_600_) of 0.1 using fresh M-H broth. Antibiotics, including piperacillin, ceftazidime, cefepime, imipenem, meropenem, aztreonam, and ciprofloxacin, were serially diluted twofold from an initial concentration of 64 μg/mL to 0.5 μg/mL. Each dilution (1 mL) was aliquoted into a 24-well plate, followed by the addition of 100 μL of the adjusted bacterial suspension (OD_600_ = 0.1). The mixtures were thoroughly mixed by pipetting, and 2(SPEEK+ AidH) was added to each well.

#### Control groups

For the negative control, we took 1 mL of M-H broth medium, added 100 μL of high-temperature inactivated bacterial solution with OD_600_ = 0.1, and mixed well. Finally, we added 2 (SPEEK+AidH).

For the positive control, we took 1 mL of M-H broth medium with concentration gradients ranging from 64 μg/mL to 0.5 μg/mL, added 100 μL of bacterial solution with OD_600_ = 0.1, mixed well, and put in 2 (SPEEK+ sodium phosphate buffer).

All plates were incubated at 37°C for 18–24 h, and the minimum inhibitory concentrations (MICs) were determined visually.

#### Statistical analysis

Experimental data were analyzed using IBM SPSS Statistics 26 software. Intergroup differences were compared using Student’s *t*-test, with statistical significance denoted as follows: *P* < 0.05 (*), *P <* 0.01 (**), and *P <* 0.001 (***).

## RESULTS AND DISCUSSION

### Optimization of adsorption time and ratio for SPEEK-loaded AidH

To determine the optimal adsorption time and ratio for immobilizing AidH onto SPEEK, the enzymatic activity of AidH@SPEEK was measured using *p*-nitrophenyl acetate as the substrate under varying adsorption durations and enzyme-to-carrier ratios. This experimental design was based on the dynamic adsorption characteristics of SPEEK, where adsorption capacity correlates positively with contact time but is constrained by the density of functional groups on the carrier surface. The results confirmed a nonlinear relationship between adsorption capacity and enzyme loading, consistent with theoretical models describing saturation adsorption on sulfonated polymer carriers (26, 27).

**Fig 1:**
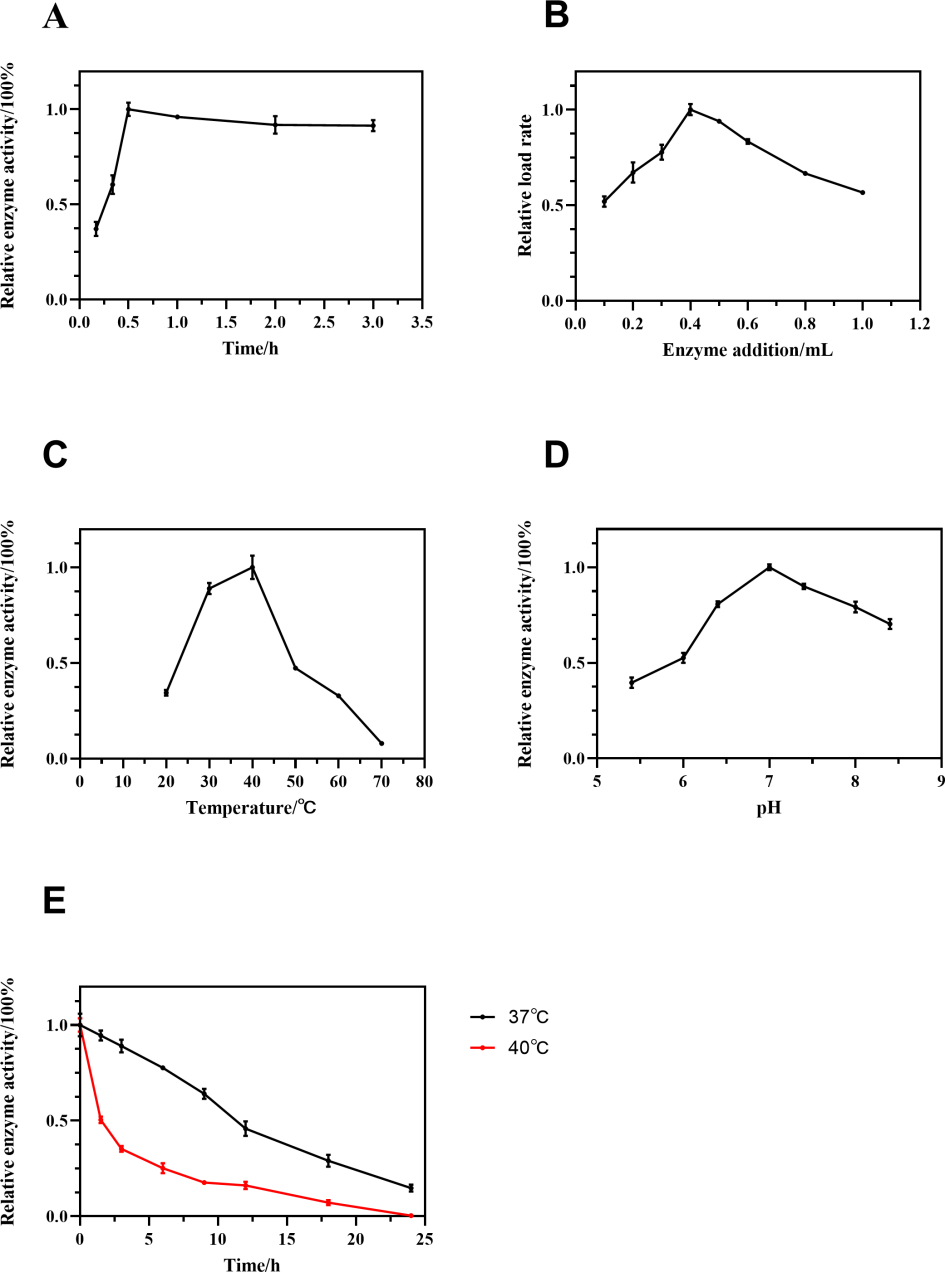
Enzymatic characterization of AidH@SPEEK. (**A**) Determination of optimal adsorption time for AidH immobilization on SPEEK. (**B**) Optimization of AidH-to-SPEEK loading ratio. (**C**) Effect of reaction temperature on AidH@SPEEK activity. (**D**) Effect of reaction pH on AidH@SPEEK activity. (**E**) Thermal stability analysis of AidH@SPEEK.

As shown in Fig. 1A, the enzymatic activity of AidH@SPEEK reached its peak after 30 min of adsorption at low temperature. Prolonging the adsorption time to 1, 2, or 3 h led to a slight decline in activity (0.91- to 0.96-fold of the 30-min value). This suggests that SPEEK’s surface active sites rapidly bind AidH during the initial phase, achieving efficient loading. Extended durations may induce conformational changes or aggregation of enzyme molecules, reducing effective loading capacity. These findings highlight the critical role of SPEEK’s surface properties in adsorption dynamics (28, 29). Additionally, low-temperature adsorption likely mitigates nonspecific interactions between the enzyme and carrier, preventing activity loss due to prolonged exposure (30, 31).

When SPEEK was loaded with increasing amounts of AidH (Fig. 1B), the relative loading efficiency peaked at 400 μL of AidH. Beyond this volume, efficiency declined, likely due to saturation of surface binding sites on SPEEK. Excess enzyme molecules may encounter steric hindrance or fail to bind effectively, reducing overall loading efficiency (31).

### Enzymatic properties of SPEEK-loaded AidH

The enzymatic activity of AidH@SPEEK was evaluated under varying temperatures and pH conditions using *p*-nitrophenyl acetate as the substrate (Fig. 1C through E). At 30–40°C, AidH@SPEEK retained over 89% of its relative activity within 4 min, demonstrating robust thermal stability in this range. The optimal temperature for activity was 40°C, aligning with reported values for YtnP, Y2-aiiA, and MomL lactonases (32, 33) but lower than those of AidB (60°C) and AaL lactonases (58.2°C) (34, 35). Compared to free AidH (21), the temperature-dependent activity profile of AidH@SPEEK differed slightly, possibly due to structural stabilization by SPEEK, which preserves catalytic conformation. The 40°C optimum may reflect enhanced stability of the enzyme-carrier complex and optimal alignment of the active site for substrate binding.

For pH dependence (Fig. 1D), AidH@SPEEK maintained >90% relative activity at pH 7.0–7.4, indicating preferential activity in neutral to mildly alkaline environments. Extreme pH values likely disrupt enzyme-substrate interactions by altering ionization states of active-site residues or the substrate itself, consistent with the behavior of most AHL lactonases (36–38). Thermal stability assays (Fig. 1E) revealed rapid activity loss at 40°C (50% residual activity at 1.5 h; complete inactivation by 24 h). In contrast, at 37°C, AidH@SPEEK retained 64% activity after 9 h and 15% after 24 h, outperforming the thermal stability of AHL lactonase AiiK (38). This underscores the composite’s enhanced stability under physiologically relevant temperatures, likely due to SPEEK’s hydrophobic surface shielding the enzyme from denaturation (39). This underscores the composite’s enhanced stability under physiologically relevant temperatures, likely due to SPEEK’s hydrophobic surface shielding the enzyme from denaturation.

SPEEK effectively immobilized AidH while preserving its catalytic function. As an AHL lactonase, AidH disrupts *Pseudomonas aeruginosa* quorum sensing by degrading signaling molecules (e.g., C4HSL and 3-oxo-C12-HSL) (21, 40). The improved thermal stability of AidH@SPEEK may arise from structural protection by SPEEK’s hydrophobic matrix, while its pH compatibility with physiological conditions enhances applicability in biological systems. These properties position AidH@SPEEK as a promising candidate for further exploration in quorum-sensing inhibition and biofilm control.

### Effect of AidH@SPEEK on PA growth

To investigate the interaction between AidH@SPEEK and PA growth, three PA strains carrying four QS-related genes and two virulence genes were selected via PCR screening: PA1 (a third-generation cephalosporin and monobactam-resistant strain), PA2 (a non-resistant strain), and PA3 (a carbapenem- and quinolone-resistant strain). The growth dynamics of PA cultures supplemented with varying amounts of AidH@SPEEK were continuously monitored.

**Fig 2:**
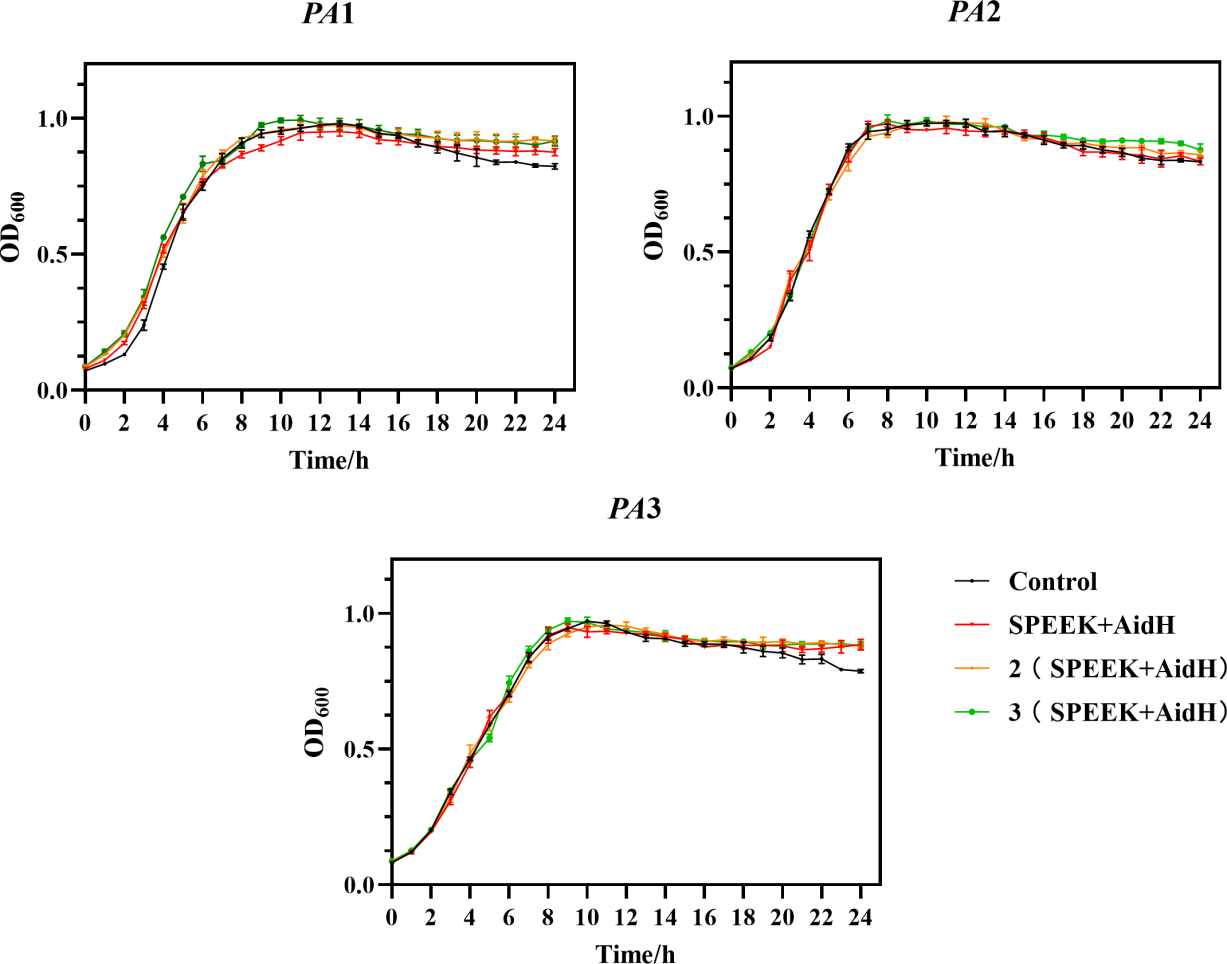
Effects of AidH@SPEEK at variable loading ratios on *Pseudomonas aeruginosa* (PA) growth.

As shown in Fig. 2, AidH@SPEEK exhibited a mild growth-promoting effect on PA1 during the logarithmic phase (2–8 h). This phenomenon might be attributed to components in AidH@SPEEK that enhance metabolic activity or create a favorable microenvironment for PA1. For instance, AidH@SPEEK may release factors stimulating cell division or improve membrane permeability, thereby facilitating nutrient uptake. In contrast, no significant growth promotion or inhibition was observed for PA2 or PA3, likely due to differences in membrane structure or metabolic pathways between strains. PA2 and PA3 might lack receptors or enzymes required to interact with bioactive components of AidH@SPEEK.

Notably, AidH@SPEEK delayed PA cell death during the decline phase (18–24 h), suggesting interference with mechanisms driving bacterial senescence. For example, AidH@SPEEK might mitigate stress responses or apoptosis triggered by nutrient depletion or metabolite accumulation, thereby reducing mortality rates in later growth stages. This could influence PA survival and potential infection persistence. Previous studies indicate that quorum quenching enzymes degrade acyl-homoserine lactones (AHLs) and alter QS-regulated phenomena in PA without affecting growth (23, 41, 42). However, QS signaling has also been reported to induce PA autolysis (43). The AidH-immobilized SPEEK composite likely disrupts QS-mediated autolysis by scavenging signal molecules. These findings highlight the complex interaction between AidH@SPEEK and PA, extending beyond simple growth modulation to include the regulation of late-stage bacterial life cycle dynamics.

### Determination of the optimal dosage of AidH@SPEEK against *Pseudomonas aeruginosa*

**Fig 3:**
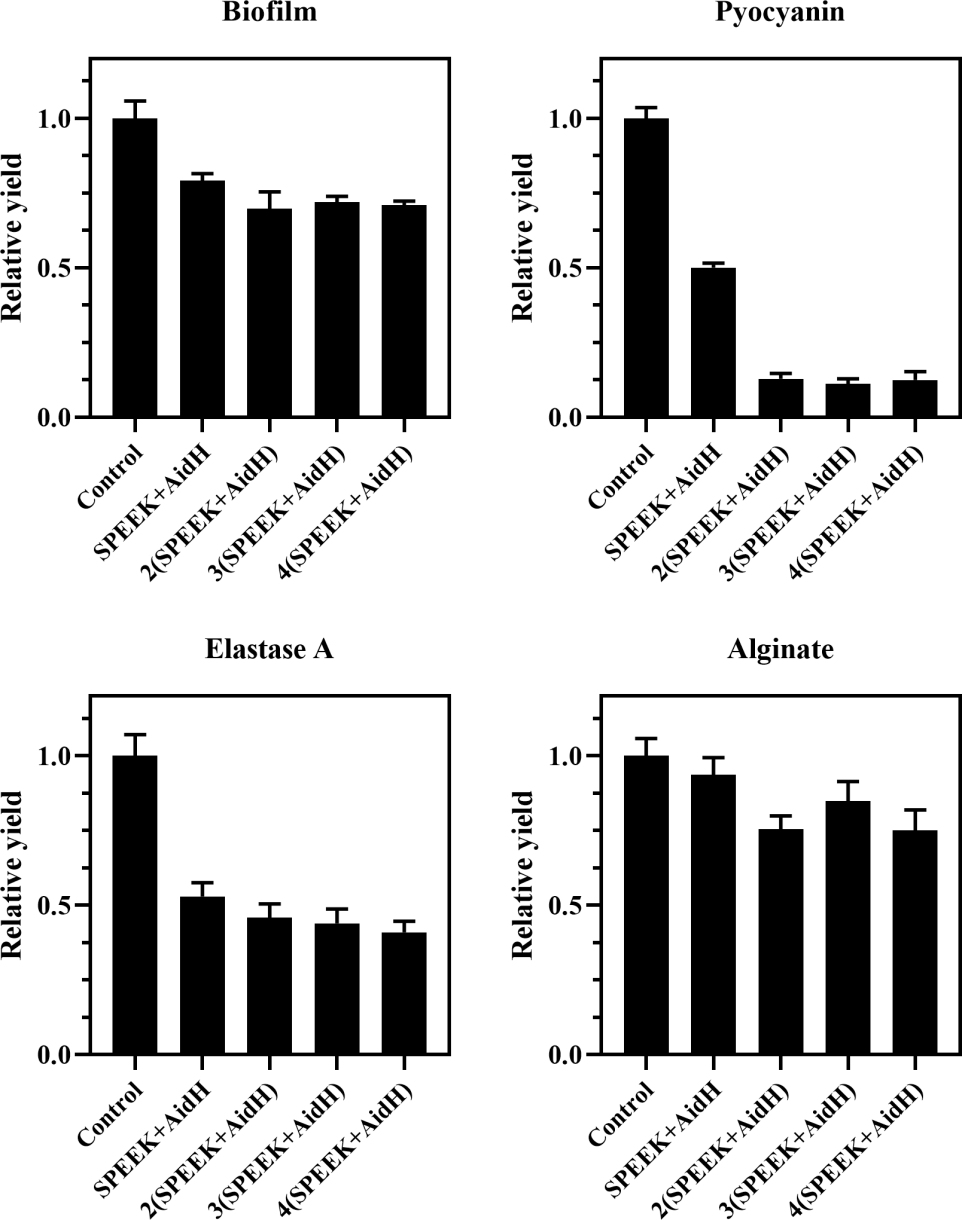
Inhibitory effects of AidH@SPEEK at variable loading ratios on biofilm formation and virulence factors in *Pseudomonas aeruginosa*.

To determine the optimal dosage of AidH@SPEEK against PA, varying amounts of the composite were supplemented into PA2 cultures. As shown in Fig. 3, biofilm formation and virulence factor production decreased proportionally with increasing AidH@SPEEK concentrations, with the optimal dosage identified as 2 (SPEEK+AidH). At this concentration, significant inhibition rates were achieved for both biofilm (30.1%) and key virulence factors. Notably, pyocyanin production was inhibited by 87.1%, indicating a potent suppression of this critical pathogenic pigment. Pyocyanin not only contributes to PA infection processes but also reflects bacterial virulence levels (44, 45), and its high inhibition rate suggests a potential reduction in PA-induced inflammatory responses. Biofilm inhibition (30.1%) surpassed the efficacy of AidC (a reference compound) reported in previous studies (23). Although lower than pyocyanin suppression, this inhibition is biologically significant as biofilms are essential for PA colonization, resistance to clearance mechanisms, and persistent infections (46). Furthermore, elastase A activity was reduced by 54.1%, which may limit PA degradation of host elastin, a key structural protein (47). Alginate production inhibition (24.5%) also implies disruption of PA physiological functions and host-pathogen interactions (48). Collectively, these results demonstrate the multi-targeted anti-virulence effects of AidH@SPEEK. The identified optimal dosage provides a critical foundation for further research and highlights the composite’s potential in attenuating PA pathogenicity through coordinated suppression of diverse virulence mechanisms.

### Strain-specific effects of AidH@SPEEK on pyocyanin secretion in *Pseudomonas aeruginosa*

**Fig 4:**
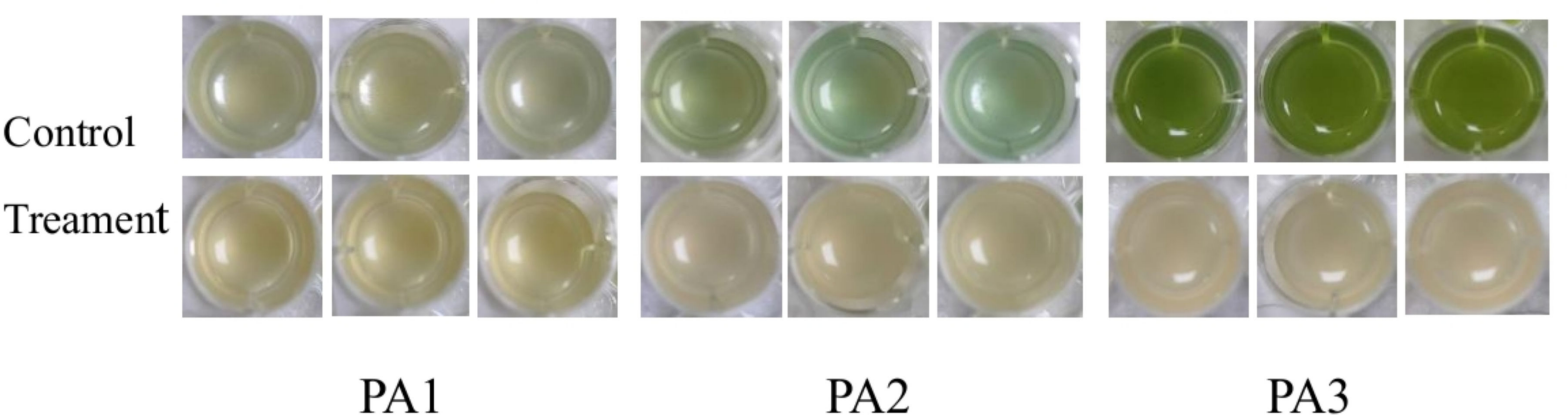
Morphological characteristics of *Pseudomonas aeruginosa* cultures following 24 h co-incubation with AidH@SPEEK.

**Fig 5:**
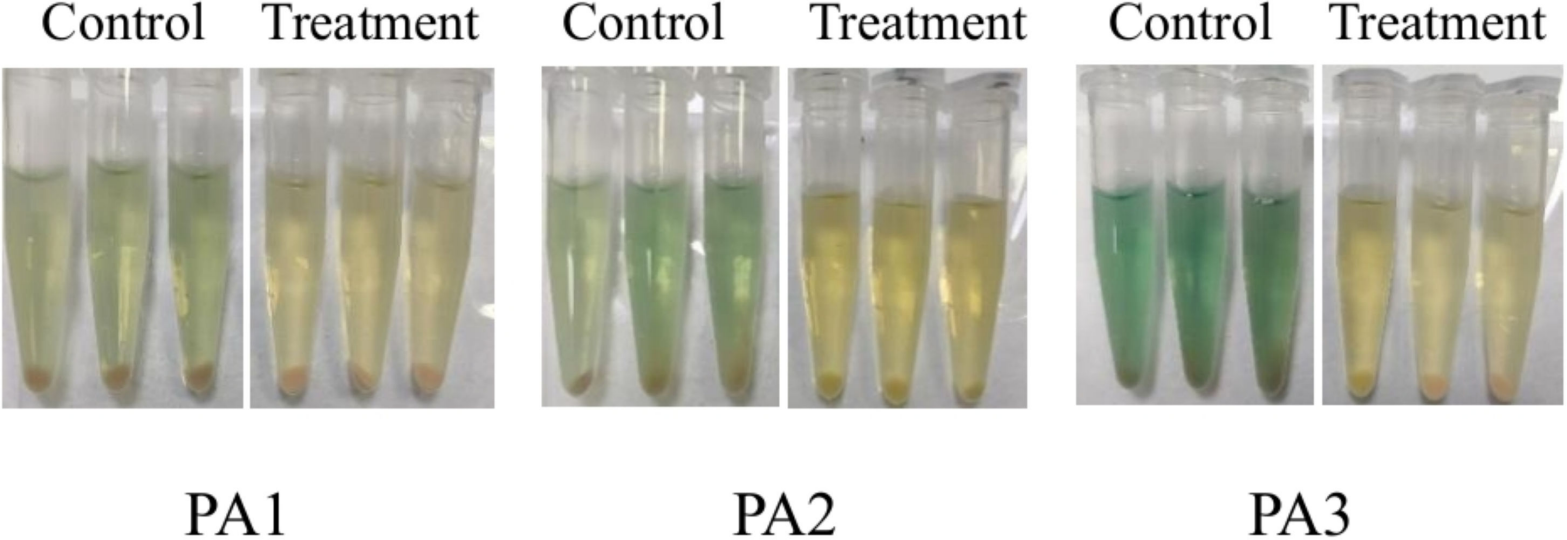
Morphological characteristics of *Pseudomonas aeruginosa* cultures post-centrifugation following 24 h co-incubation with AidH@SPEEK.

**Fig 6:**
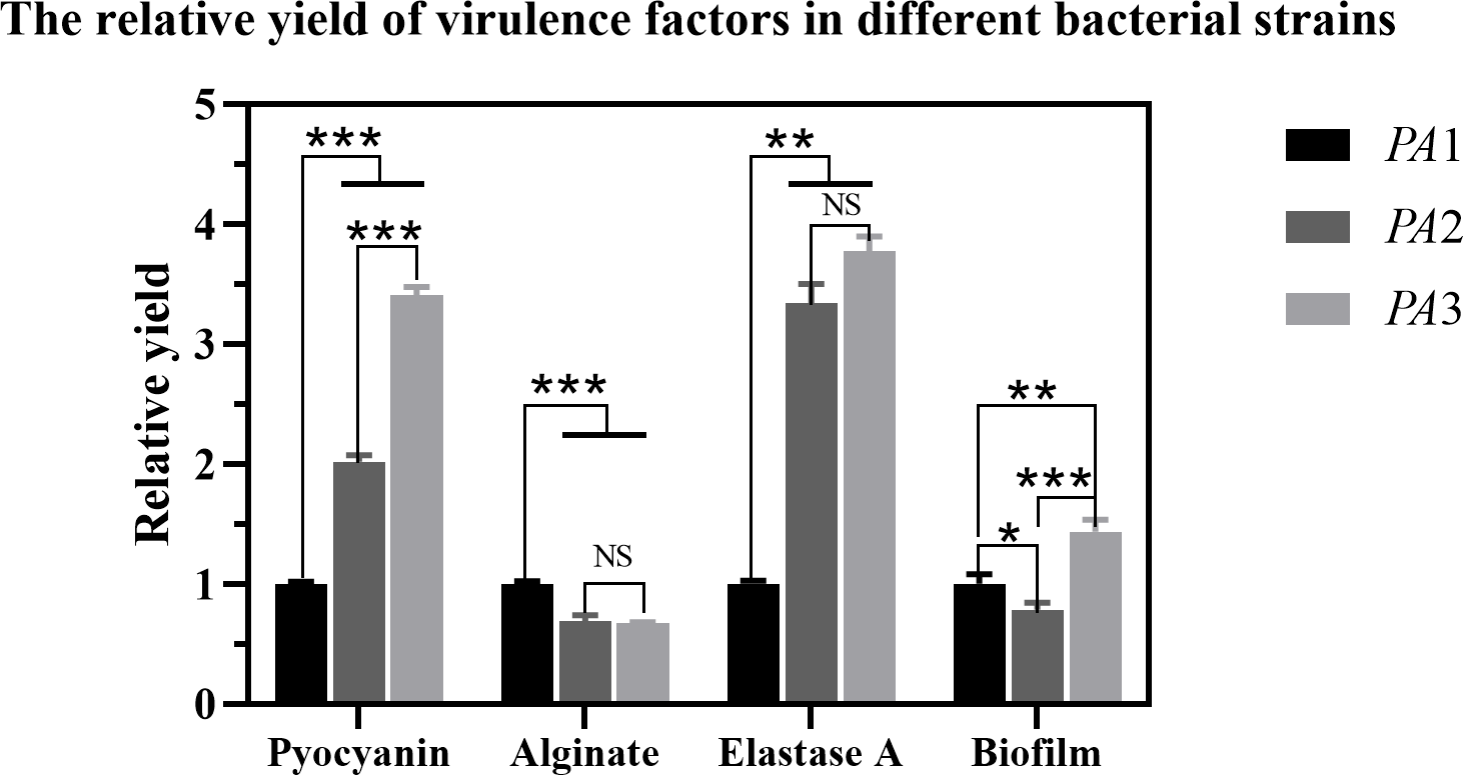
Biofilm formation and virulence factor yield profiles in three clinical *Pseudomonas aeruginosa* isolates. *P < 0.05; **P < 0.01; ***P < 0.001; and NS (not significant), *P* ≥ 0.05.

Experiments were conducted using the three aforementioned PA strains. As shown in Fig. 4 and 5, distinct variations in pyocyanin secretion were observed among the three PA strains after 24 h of culture, with significant differences between experimental (AidH@SPEEK-treated) and control groups. These results indicate that the inhibitory effects of AidH@SPEEK on pyocyanin production are strain-specific, potentially attributable to differences in genetic regulation or metabolic pathways across PA strains (49, 50). Through the detection of the biofilm and virulence factor secretion amounts of three PA control strains, the results are shown in Fig. 6. There are significant differences among different PA strains in terms of biofilm yield and virulence factor secretion amounts. Such differences may lead to different pathological results and treatment difficulties during the actual infection process.

**Fig 7:**
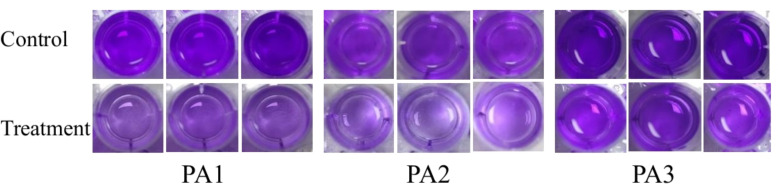
Crystal violet staining assay for biofilm quantification.

**Fig 8:**
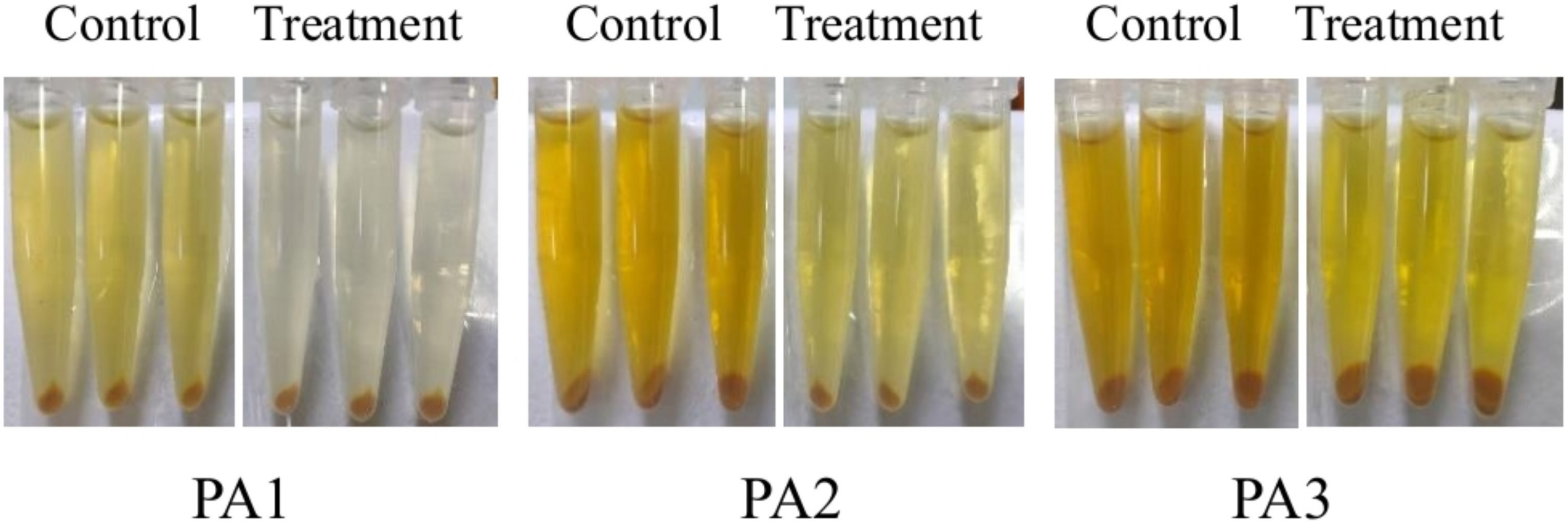
Assay results of elastase A.

**Fig 9:**
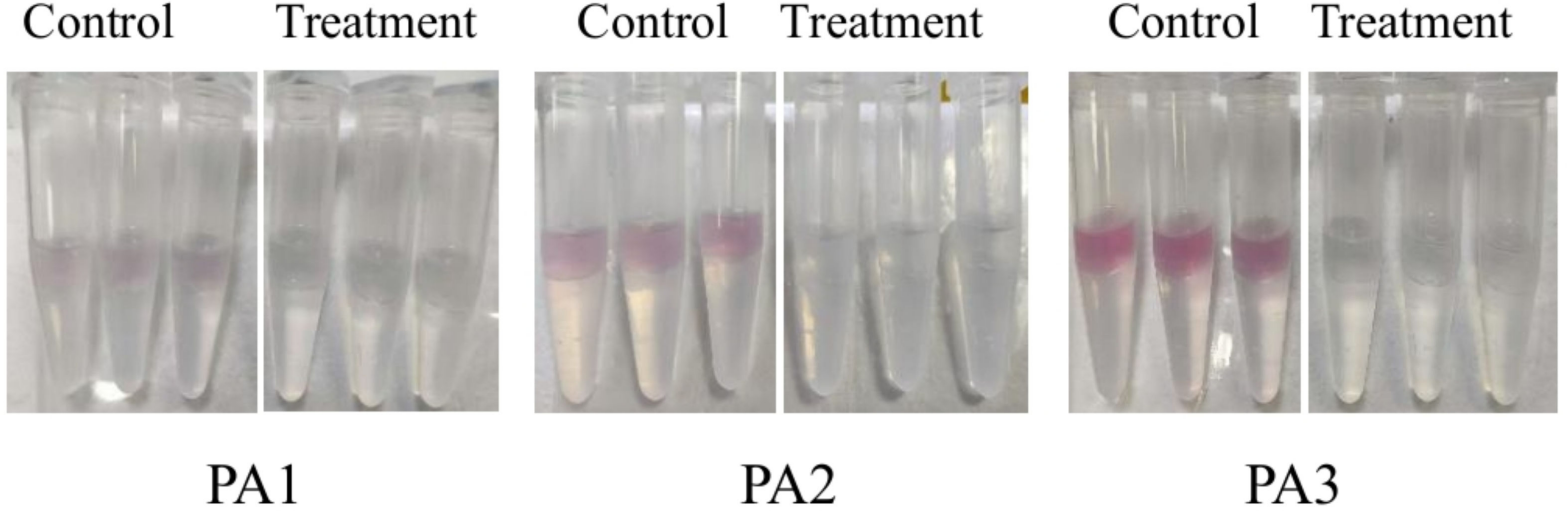
Results of pyocyanin determination.

**Fig 10:**
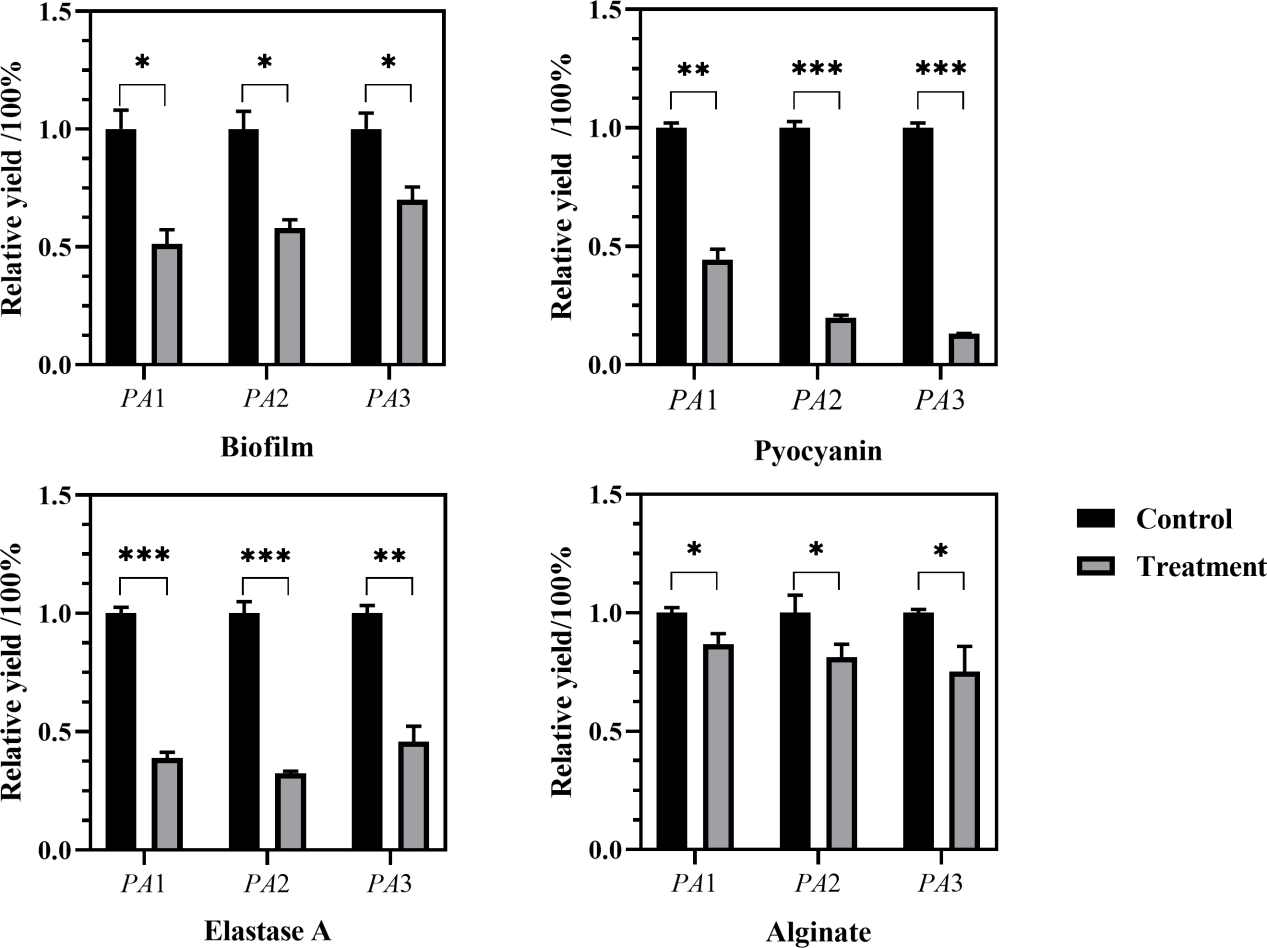
The effects of AidH@SPEEK on PA biofilms and virulence factor formation. *P < 0.05, **P < 0.01, and ***P < 0.001.

Figures 7 to 9 show the appearance images of the measurement results of biofilm, *Pseudomonas aeruginosa*, and elastase A (as butyl acetate in the alginate detection reagent is corrosive to the plastic plate, no photos were taken). The specific data statistics results are shown in Fig. 10. In the comparison between the experimental group and the control group, the biofilm production amounts of PA1, PA2, and PA3 in the experimental group, the production amounts of *Pseudomonas aeruginosa*, elastase A, and alginic acid were significantly lower than in the control group. Among them, the inhibitory effects of *Pseudomonas aeruginosa* and elastin were similar to those of MBP-AhlS and zeolite 4A. However, the inhibitory effect of the biofilm is significantly inferior to that of zeolite 4A (51, 52), and biofilm suppression was markedly less effective than that of zeolite 4A. Strain-dependent inhibition variations were evident: PA3 showed the highest pyocyanin inhibition (87.0%), whereas PA1 exhibited the lowest alginate suppression (13.1%). These disparities suggest differential responsiveness to AidH@SPEEK, potentially mediated by strain-specific QS interference efficiency or intracellular signaling pathway modulation. Collectively, AidH@SPEEK exhibited broad-spectrum anti-virulence activity against multiple PA strains, albeit with variable efficacy across targets. These findings underscore its potential for tailored anti-infective strategies, emphasizing the need for strain-specific optimization when deploying this composite to disrupt PA pathogenicity.

### Regulation of quorum-sensing and virulence gene expression by AidH@SPEEK

**Fig 11:**
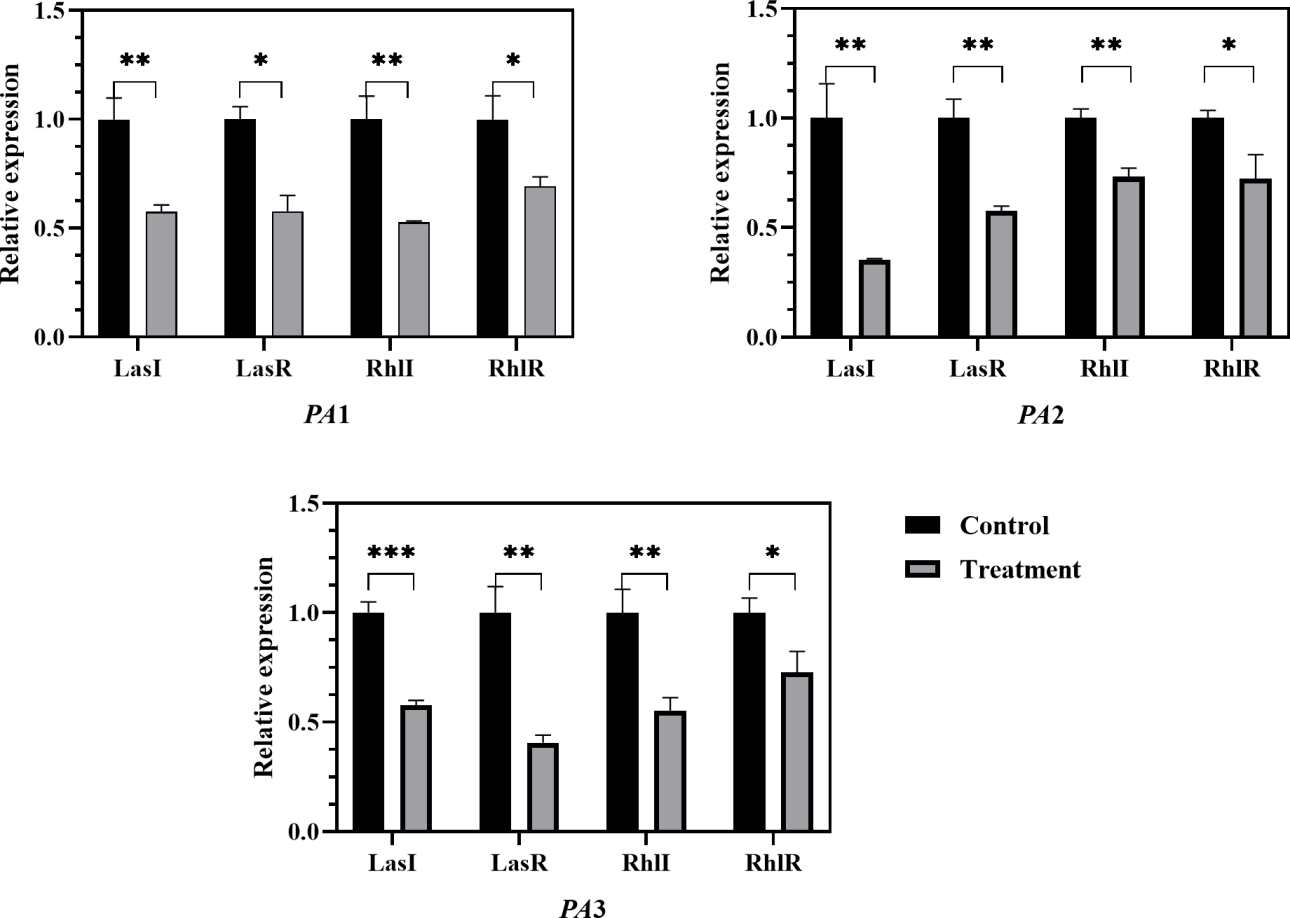
The regulatory effect of AidH@SPEEK on quorum-sensing genes. *P < 0.05, **P < 0.01, and ***P < 0.001.

**Fig 12:**
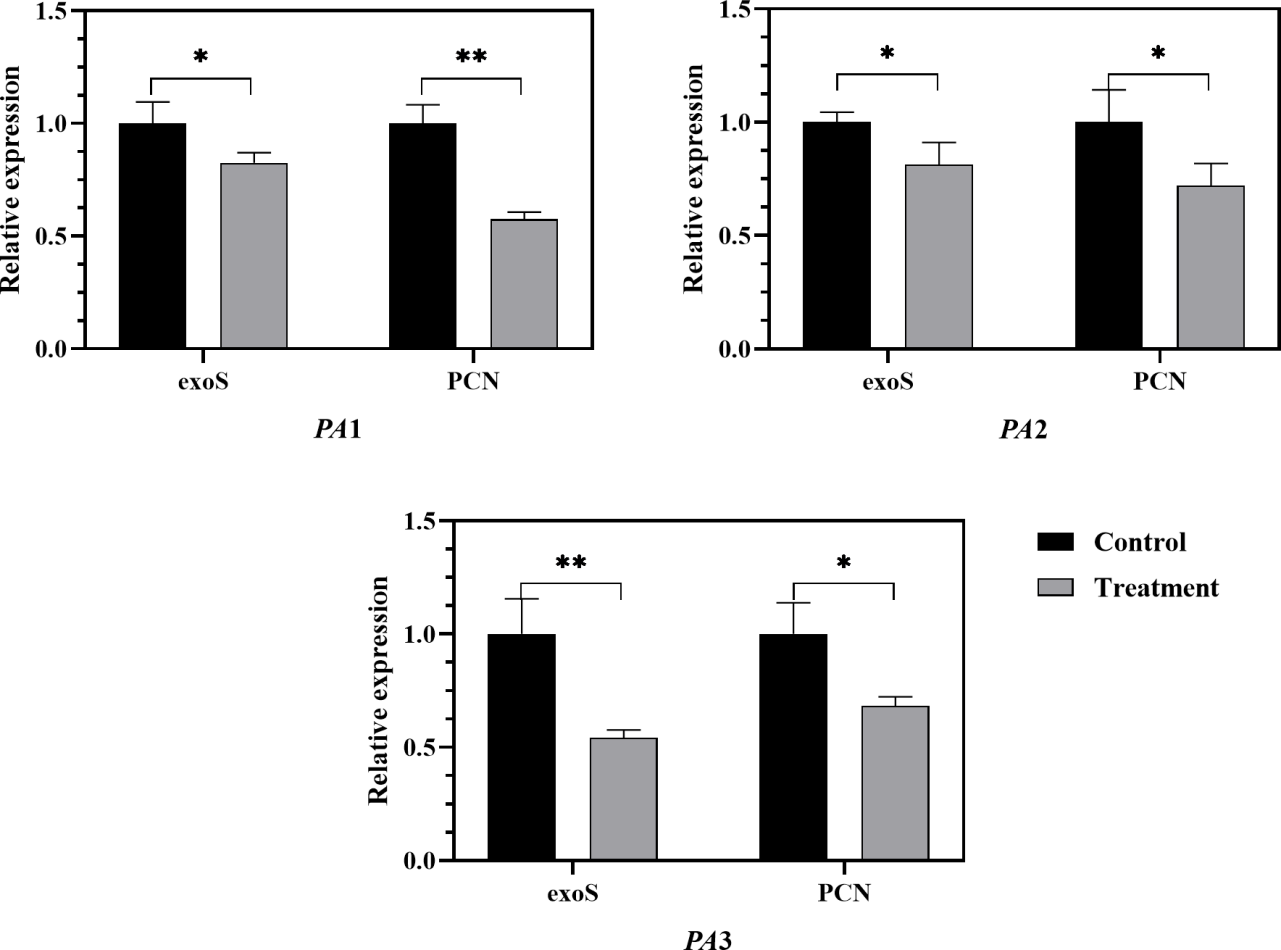
The regulatory effect of AidH@SPEEK on virulence genes. *P < 0.05 and **P < 0.01.

To confirm the inhibitory effect of AidH@SPEEK on quorum-sensing and virulence genes expression, the experiment examined the Las system and Rhl system mediated by N -acyl homoserine lactone signal molecules and the expression of relevant genes LasR/LasI, RhlR/RhlI, and virulence genes exoS and phzM. As shown in Fig. 11 and 12, after the action of 2 (SPEEK + AidH), the expression of quorum-sensing genes LasR/LasI and RhlR/RhlI and virulence genes exoS and phzM was downregulated. Studies have shown that exoS encodes the bifunctional toxin ExoS, which disrupts the host cell cytoskeleton with ADP-ribosyltransferase activity, inhibits the phagocytic function of immune cells, and induces neutrophil apoptosis, and its expression is directly regulated by the QS system, particularly the Las and Rhl systems (53, 54). Numerous studies have demonstrated a strict positive correlation between phzM transcriptional activity and the pyocyanin-producing capacity of *Pseudomonas aeruginosa*; consequently, quantifying phzM mRNA has become a routine molecular proxy for the organism’s virulence potential (55, 56). For example, Song et al. evaluated pyocyanin synthesis by measuring phzM expression while investigating the interplay between bacterial motility and virulence (55). Analogously, synthetic biology efforts have achieved substantial increases in pyocyanin titer through targeted optimization of the phz operon—of which phzM is an essential component. Building on this consensus, we selected phzM as the key biomarker to assess the impact of the SPEEK@AidH complex on pyocyanin biosynthesis. AidH is a metal-ion-independent AHL lactonase whose active center can hydrolyze the lactone ring of AHL signal molecules. When AidH combines with SPEEK to form a complex, its stability is enhanced, allowing for sustained degradation of AHL molecules in the microenvironment and blocking the binding of LasR and RhlR receptors to signal molecules (57).

The downregulation of quorum-sensing gene expression by AidH@SPEEK suggests that it may interfere with the normal intercellular signaling mechanism of PA, thereby weakening its ability to initiate programs related to virulence factor synthesis and biofilm formation from the source. Consequently, a series of subsequent quorum behaviors is inhibited. This is in line with the previous experimental results of biofilm and virulence factor formation inhibition. It further reveals the potential molecular mechanism of AidH@SPEEK, that is, it exerts its effects by regulating the expression of quorum-sensing genes, thereby inhibiting various pathogenic-related characteristics of PA. This provides key insights into the antibacterial and disease-preventing principles of AidH@SPEEK and offers new ideas for the development of potential quorum-sensing-based antibacterial drugs.

### Synergistic effects of AidH@SPEEK and antibiotics

*Pseudomonas aeruginosa* often exists in a biofilm state, showing strong tolerance to various antibiotics and immune defenses (58, 59). Due to the uncontrolled and excessive use of conventional antibiotics, there is an over-selection pressure in clinical settings, leading to multidrug resistance. Currently, *P. aeruginosa* has developed resistance to many antibiotics, including aminoglycosides, quinolones, and β-lactams (60). Studies have shown that YtnP lactonase exhibits a synergistic effect when combined with gentamicin and an additive effect when combined with imipenem. This indicates that YtnP lactonase can potentially reduce antibiotic doses and enhance their therapeutic efficiency when used with antibiotics (61).

As presented in Table 2, the positive control (-1) and the experimental group (-2) are shown. It should be noted that the negative control, which was treated with heat-inactivated bacteria, showed no discernible bacterial growth, as indicated by constant OD_600_ readings over time. Consequently, this group was excluded from the final data presentation in Table 2, and only the positive control was utilized as the baseline for comparison. For PA strains with different resistance profiles, AidH@SPEEK, when combined with multiple clinical antibiotics, shows varying effects. For PA1 (a strain resistant to third-generation cephalosporins and monobactams), the MIC of ceftazidime, cefepime, imipenem, and aztreonam is reduced to different degrees. This indicates that AidH@SPEEK enhances PA1’s susceptibility to these antibiotics. This could be because AidH@SPEEK disrupts the bacterial protective barrier by inhibiting PA biofilm formation, allowing antibiotics to more easily reach their targets. Alternatively, it might alter some physiological states or molecular mechanisms of PA, increasing the bactericidal effect of antibiotics. This reduces the required antibiotic concentration for effective inhibition or killing. It provides a new approach for treating PA infections and managing antibiotic resistance. However, the specific mechanisms of how AidH@SPEEK interacts with different antibiotics need further study to optimize combination therapy and improve clinical outcomes.

**TABLE 2:** The combination effect of AidH@SPEEK with antibiotics^*a*^

Antibiotic	PA1-1 MIC (μg/mL)	PA1-2 MIC (μg/mL)	PA2-1 MIC (μg/mL)	PA2-2 MIC (μg/mL)	PA3-1 MIC (μg/mL)	PA3-2 MIC (μg/mL)
Piperacillin	4	4	32	4	4	4
Ceftazidime	64	2	8	2	2	2
Cefepime	8	4	2	2	16	2
Imipenem	2	1	1	1	1	1
Meropenem	1	1	2	1	8	1
Aztreonam	64	32	2	2	16	2
Ciprofloxacin	1	1	0.5	0.5	4	0.5

^*a*^ “-1” represents the positive control group. “-2” represents the experimental group.

### Conclusion

*Pseudomonas aeruginosa* biofilms are highly antibiotic resistant. In this study, we successfully developed a stable and efficient AidH@SPEEK composite. The optimal loading conditions are 400 μL of AidH enzyme and 30 min of loading time, with an optimal reaction temperature of 40°C and pH of 7. Notably, it shows better enzyme activity stability at 37°C. AidH@SPEEK can substantially suppress PA biofilm formation and secretion of virulence factors and downregulate quorum-sensing genes (LasR/LasI, RhlR/RhlI) and virulence genes (exoS, phzM). It also enhances antibiotic susceptibility. These findings offer novel strategies for preventing and treating *P. aeruginosa* infections.

### Limitations and future perspectives

This study has successfully established the optimal immobilization conditions and demonstrated enhanced short-term operational stability of the AidH enzyme immobilized on the SPEEK carrier. However, it is crucial to acknowledge the current limitations and outline the necessary future directions to fully assess the translational potential of the SPEEK@AidH system.

A primary focus for subsequent investigation is the evaluation of long-term stability beyond the 24-h period examined here. The operational stability of an immobilized enzyme under prolonged operational conditions is a critical determinant of its economic viability for continuous industrial bioprocesses or extended batch reactions. While our data indicate promising stability at 37°C within a 24-h frame, a comprehensive assessment over extended durations is essential to determine the functional half-life of the biocatalyst. Such studies are vital for quantifying the inevitable gradual inactivation processes, which may arise from enzyme leaching, conformational denaturation, or cumulative mechanical degradation of the support matrix.

In this context, it is important to clarify the role of the free AidH group in the present study. This group was primarily employed as a process optimization control to assess immobilization efficiency rather than as an essential comparator for direct efficacy evaluation. While the inclusion of a free enzyme control would undoubtedly provide a more comprehensive basis for evaluating the immobilization process—allowing direct comparison of activity recovery and immediate protective benefits—such a comparison was beyond the central scope of this work, which focused on demonstrating the overall catalytic performance and initial operational robustness of the SPEEK@AidH complex. We fully acknowledge this conceptual and methodological limitation. Therefore, future studies should directly compare the operational half-life and long-term stability of SPEEK@AidH with an equivalent amount of free AidH under identical conditions. Such comparative data are indispensable for precisely quantifying the stabilization advantage conferred by the SPEEK matrix.

Furthermore, investigating long-term stability would facilitate a more rigorous comparison with the free enzyme, unequivocally demonstrating the protective advantage conferred by the SPEEK immobilization matrix. Future work should aim to quantify key performance metrics such as the total turnover number (TTN) or the number of operational cycles achievable before significant activity loss occurs. These data are indispensable for a robust techno-economic analysis of the process. Ultimately, elucidating the long-term performance is a fundamental step toward transitioning this promising biocatalytic system from a proof-of-concept stage into a scalable and sustainable technology.

Concurrently, the potential biomedical application of SPEEK@AidH, particularly in implant-associated infection control, necessitates a thorough evaluation of its biocompatibility and safety profile—a limitation of the present work. As rightly noted, the biomedical potential of this functionalized material must be rigorously validated through standardized *in vitro* and *in vivo* assessments prior to any clinical consideration. Future studies must include systematic biocompatibility evaluations, such as *in vitro* cytotoxicity assays using relevant mammalian cell lines (e.g., fibroblasts L929, macrophages RAW 264.7) to quantify cell viability and inflammatory responses upon exposure, and hemocompatibility tests, specifically hemolysis assays, to ensure the material does not induce red blood cell lysis. Moreover, the efficacy of SPEEK@AidH in modulating infection dynamics must be confirmed in a physiologically relevant environment. Employing established animal models of implant-associated infection (e.g., a subcutaneous murine model with an implanted SPEEK disk) will be indispensable for investigating the material’s capacity to mitigate biofilm formation, reduce bacterial burden, and modulate host immune responses *in situ* while monitoring for any adverse local or systemic effects.

Addressing these aspects—long-term operational stability and comprehensive biocompatibility—will be the focus of our ongoing research. We concur that these studies are not merely supplementary but are crucial and sequential steps in translating the SPEEK@AidH system from a promising *in vitro* construct into a viable and safe therapeutic strategy.
